# Genome-Wide Identification and Analysis of *R2R3-MYB* Genes Response to Saline–Alkali Stress in Quinoa

**DOI:** 10.3390/ijms24119132

**Published:** 2023-05-23

**Authors:** Yuqi Liu, Mingyu Wang, Yongshun Huang, Peng Zhu, Guangtao Qian, Yiming Zhang, Lixin Li

**Affiliations:** Key Laboratory of Saline-Alkali Vegetation Ecology Restoration, Ministry of Education, College of Life Sciences, Northeast Forestry University, Harbin 150040, China

**Keywords:** transcription factors, CqMYB2Rs, stress response, metabolism, transcriptome analysis

## Abstract

Soil saline–alkalization inhibits plant growth and development and seriously affects crop yields. Over their long-term evolution, plants have formed complex stress response systems to maintain species continuity. R2R3-MYB transcription factors are one of the largest transcription factor families in plants, widely involved in plant growth and development, metabolism, and stress response. Quinoa (*Chenopodium quinoa* Willd.), as a crop with high nutritional value, is tolerant to various biotic and abiotic stress. In this study, we identified 65 R2R3-MYB genes in quinoa, which are divided into 26 subfamilies. In addition, we analyzed the evolutionary relationships, protein physicochemical properties, conserved domains and motifs, gene structure, and cis-regulatory elements of CqR2R3-MYB family members. To investigate the roles of CqR2R3-MYB transcription factors in abiotic stress response, we performed transcriptome analysis to figure out the expression file of *CqR2R3-MYB* genes under saline–alkali stress. The results indicate that the expression of the six *CqMYB2R* genes was altered significantly in quinoa leaves that had undergone saline–alkali stress. Subcellular localization and transcriptional activation activity analysis revealed that CqMYB2R09, CqMYB2R16, CqMYB2R25, and CqMYB2R62, whose *Arabidopsis* homologues are involved in salt stress response, are localized in the nucleus and exhibit transcriptional activation activity. Our study provides basic information and effective clues for further functional investigation of CqR2R3-MYB transcription factors in quinoa.

## 1. Introduction

Soil salinization and alkalization inhibit plant growth and development and seriously affect crop yields. The increasing population worldwide has led to an increasing demand for food. Therefore, improving saline–alkali land to become reserve farmland has profound significance, which is beneficial for food security and ecological security, as well as sustainable development of agriculture [1,2]. Saline–alkali stress results in ion toxicity and osmotic stress, as well as inhibition of nutrient absorption [3]. These effects lead to disrupted metabolism and ion homeostasis, impaired photosynthesis, and subsequent severely delayed plant growth and development [4,5,6]. Over their long-term evolution, plants have gained complex stress response systems to preserve the continuity of the species.

As part of the transcription factors that participate in stress regulatory network in plants, MYB proteins are one of the largest families related to abiotic stress responses in plants [7,8,9]. The MYB family is characterized by a highly conserved MYB_DNA-binding domain, which generally consists of up to four repeats (R) composing about 52 amino acids (aa). Each repeat contains three α–helices. The second and third helix of each repeat build a helix–turn–helix (HTH) structure with three regularly spaced tryptophan (or hydrophobic) residues, forming a hydrophobic core in the 3D HTH structure [10]. According to number of the repeats, MYB TFs are divided into 1R-MYB, R2R3-MYB, 3R-MYB, and 4R-MYB [11]. R2R3-type MYB proteins are the largest MYB subfamily related to abiotic stress responses (e.g., drought, dehydration, heat, and salinity) in plants [12].

Many studies have documented that plant R2R3-MYB genes regulate abiotic stress response by thickening leaf cuticular waxes, controlling stomatal aperture, and regulating the ABA signaling pathway [13,14,15]. For example, *OsMYB2*, a R2R3-MYB TF, is involved in various abiotic stress in rice. *OsMYB2*-overexpressing plants were more tolerant to salt, cold, and dehydration stresses, and more sensitive to ABA than wild-type plants [16]. AtMYB44 positively regulates ABA-induced stomatal closure and inhibits the expression of protein phosphatase 2C (PP2C) in *Arabidopsis* [17]. SbMYBHv33 negatively regulates salt tolerance, ion and ROS homeostasis, and sorghum biomass [18]. R2R3-MYB TFs have been demonstrated to regulate secondary metabolism (e.g., biosynthesis of flavonoids) and cell wall formation [19,20]. EbMYBP1 is clarified to regulate the accumulation of flavonoids in *Erigeron breviscapus*. It can increase the total flavonoid contents in plants by binding to the promoters of flavonoid biosynthesis genes to activate their expression [21]. *Arabidopsis* MYB4 and its homologues MYB7 and MYB32 interact with bHLH TFs TT8, GL3, and EGL3, thereby interfere with the transcriptional activation activity of the MBW complex. In addition, MYB4 can also inhibit the accumulation of flavonoids by inhibiting the expression of *Arogenate dehydratase 6* (ADT6), which catalyzes flavonoid biosynthesis [22]. Overexpression of *MYB6* in transgenic poplar promotes the accumulation of anthocyanins and proanthocyanidins. It also interacts with KNAT7 to inhibit the development of secondary cell walls [23].

Quinoa (*Chenopodium quinoa* Willd.) is an annual dicotyledonous herbaceous crop of the *Amaranthaceae* family [24]. Quinoa is considered to be a complete food because it is rich in all essential amino acids with a good balance, and also contains a variety of vitamins, a large number of minerals, unsaturated fat acids, dietary fiber, and is free of gluten and cholesterol. Moreover, quinoa contains numerous secondary metabolites with broad spectra of bioactivities. In the past 40 years, at least 193 secondary metabolites in quinoa have been identified, including flavonoids, phenolic acids, terpenoids, steroids, and nitrogen-containing compounds [25]. Compared with widely cultivated staple crops such as rice, wheat and corn, the nutritional composition of quinoa makes it a leader in healthy foods. On the other hand, quinoa exhibits high tolerance to adverse climate and soil conditions such as drought, salinity, and frost. For example, quinoa can adapt to drought environment through its high water-use efficiency [26]. The high tolerance to harsh environment makes it a favorable candidate for agronomic expansion [27,28,29,30]. Therefore, identification and application of quinoa candidate genes for improving stress resistance in marginal lands is very meaningful, and this has further enhanced the position of quinoa in global foods.

In this study, we identified the *R2R3-MYB* family genes in quinoa, and performed a comprehensive analysis including phylogenetic tree, gene structure, and motif composition. Transcriptome analysis revealed the expression profiles of *CqR2R3-MYB* family genes in quinoa leaves that had undergone saline–alkali stress. Our study provides basic information and valuable clues for future investigations aiming at the functional characterization of the CqR2R3-MYB genes and can be utilized in the genetic improvement of quinoa.

## 2. Results

### 2.1. Genome-Wide Identification of R2R3-MYB Family Genes in Quinoa

**Figure 1:**
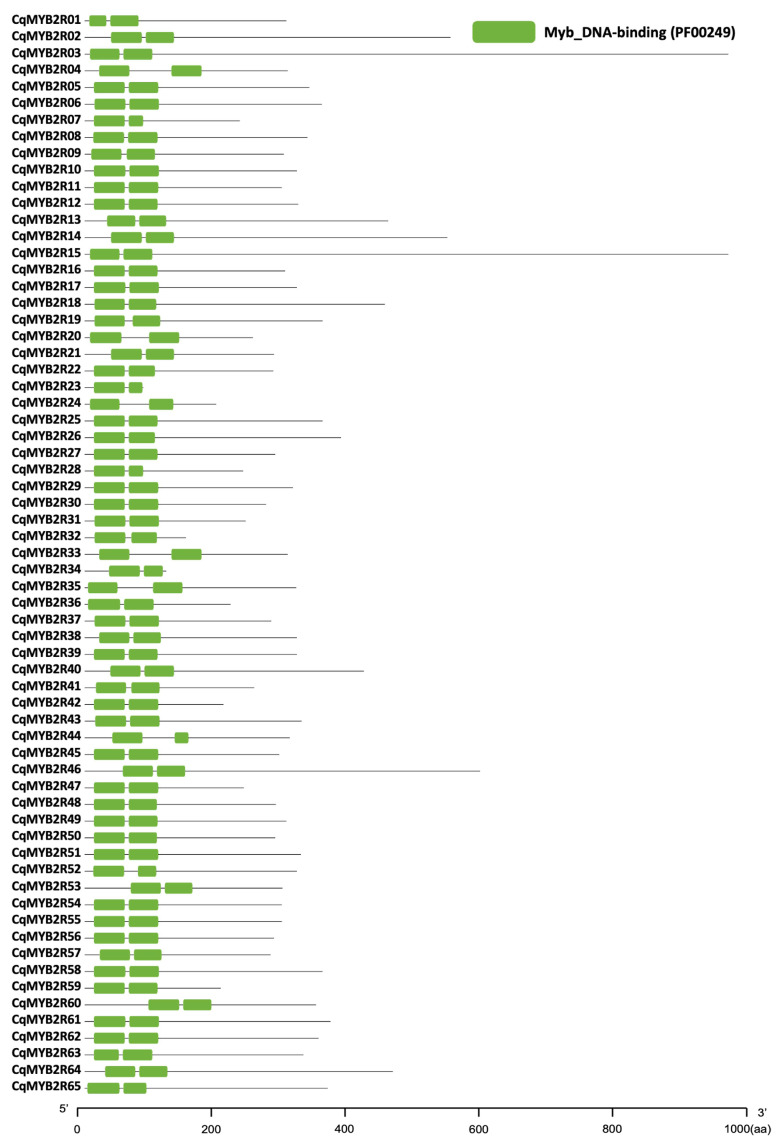
Conservative domain analysis of CqR2R3-MYB proteins. The green box represents MYB_DNA binding domain.

MYB transcription factors (TFs) can be divided into four classes based on the number of adjacent repeats (one, two, three, or four). In this study, we screened 204 MYB proteins according to the Hidden Markov Model (HMM) profile (PF00249) [11,31] in quinoa (*Chenopodium quinoa* Willd.). From them, we identified 65 R2R3-MYB proteins depending on the adjacent repeating sequences and named them as CqMYB2R1-CqMYB2R65 according to the Gene IDs. The CqR2R3-MYB proteins harbor two adjacent repetitive MYB_DNA binding domains (R) at N-terminus (Figure 1). Their basic information of CqR2R3-MYB TFs is summarized in Table 1. The average protein length is 336 amino acid (aa) residues, the average molecular weight is 37.8 kDa, and most CqR2R3-MYBs belong to acidic proteins with pI < 7. Prediction of subcellular localization indicates that most CqR2R3-MYB proteins are localized in the nucleus, except for CqMYB2R35, with a dual-localization of nucleus and cytoplasm.

### 2.2. Phylogenetic Analysis of CqR2R3-MYB Family Members

**Figure 2:**
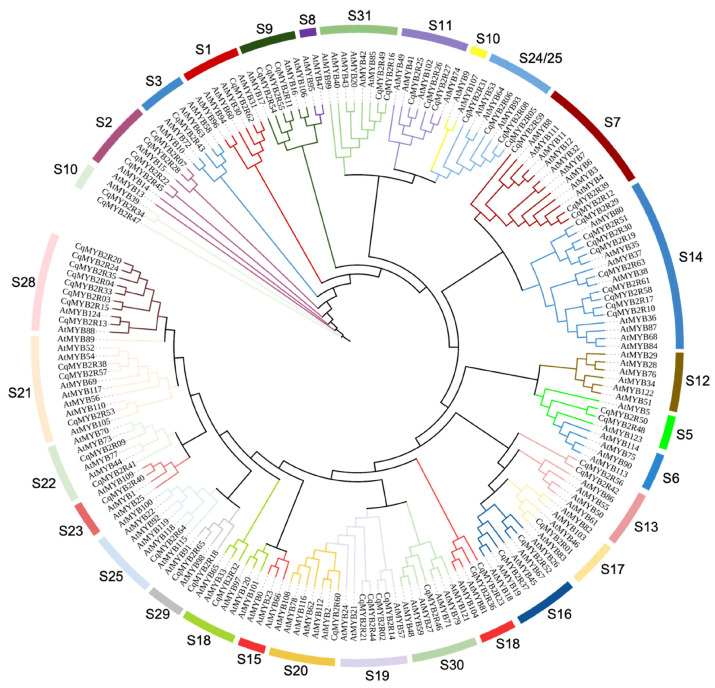
Phylogenetic analysis of R2R3-MYB proteins. A maximum-likelihood phylogenetic tree containing 65 CqR2R3-MYB proteins in quinoa and 126 in *Arabidopsis thaliana*. *At*, *Arabidopsis thaliana*; *Cq*, *Chenopodium quinoa*. The subgroups were distinguished by different colors. S1, subgroup 1.

To assess the evolutionary relationship of R2R3-MYB TFs in *Chenopodium quinoa* Willd. and *Arabidopsis thaliana* (L.), the phylogenetic tree of R2R3-MYB families was constructed using protein sequences of AtR2R3-MYBs [32] and CqR2R3-MYBs. Based on the phylogenetic tree, the AtR2R3-MYB and CqR2R3-MYB family members were divided into 31 subfamilies (Figure 2). CqR2R3-MYB proteins are found in 26 subfamilies, except for S6, S8, S10, S12, and S15 subfamilies, which only contain AtR2R3-MYBs.

### 2.3. Primary Structures of Genes and Proteins of CqR2R3-MYBs

**Figure 3:**
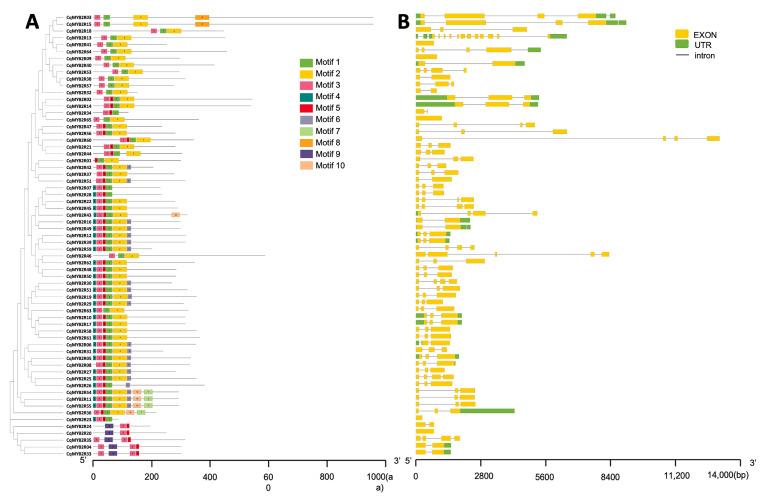
Analysis of conservative motifs and gene structure of CqR2R3-MYB family members. (**A**) Conservative motif analysis of CqR2R3-MYB proteins. Different colors represent different motifs. (**B**) Gene structure of *CqR2R3-MYBs*. UTR, untranslated region.

To gain more insight into the evolutional and structural diversity of CqR2R3-MYBs, we analyzed conserved motifs in CqR2R3-MYB proteins using the MEME suits. A total of 10 distinct and highly conserved motifs were captured (Figure 3A; Supplementary Figure S1). Motifs 1, 3, and 9 are identified as MYB domains, whereas the function of the other motifs (2, 4, 5, 6, 7, 8, and 10) is unknown. The motif distribution pattern in most CqR2R3-MYB proteins is highly conserved, which contains motif 1, motif 2, motif 3, and motif 5. In contrast, some motifs displayed specificity; for example, motif 8 only appears in CqMYB2R05 and CqMYB2R13, and motif 9 only appears in CqMYB2R04, CqMYB2R20, CqMYB2R24, CqMYB2R33, and CqMYB2R35, all of which belong to S28 (Figure 2 and Figure 3A). To further explore the structural diversity of *CqR2R3-MYB* genes, the intron–exon organization of each gene was analyzed. As shown in Figure 3B, the exon number varied from 1 to 12, and most of the CqR2R3-MYB genes have 2–4 exons, except for 4 genes having 1 exon and CqMYB2R13 having 12 exons.

### 2.4. Cis-Acting Elements in Promoters of CqR2R3-MYB Genes

**Figure 4:**
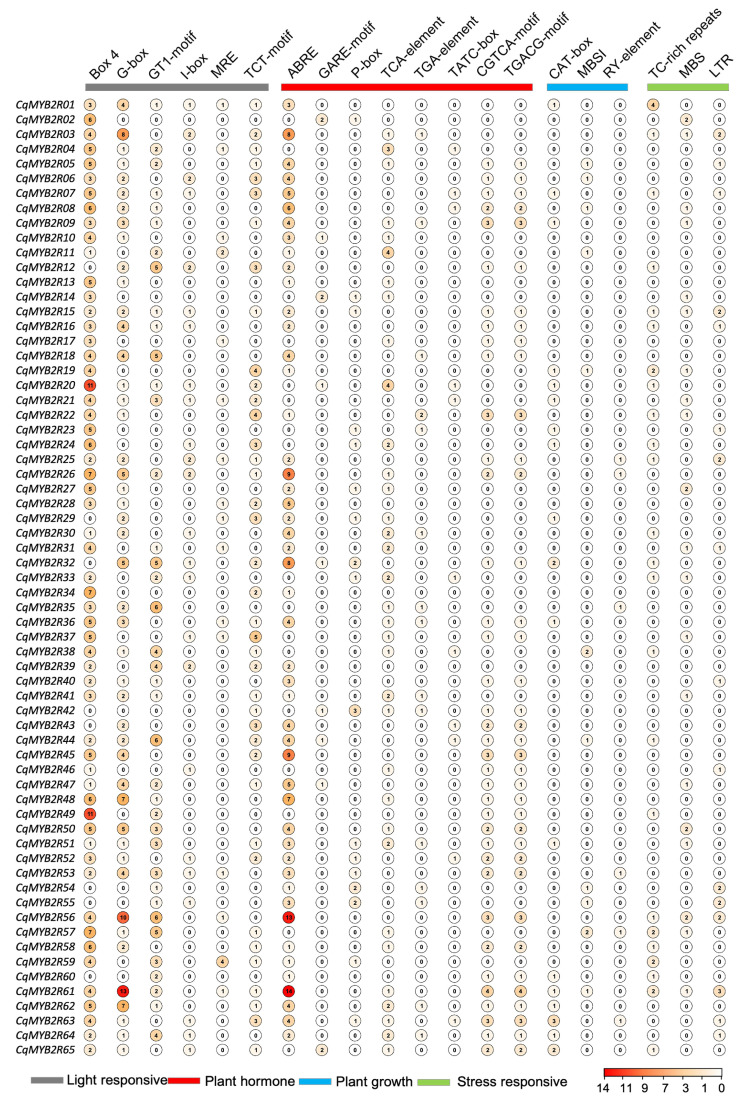
Analysis of *cis-acting* elements in *CqR2R3-MYB* promoters. The categorized groups are indicated by color bars. The score which indicates the occurrence frequency of each *cis-acting* element in each promoter is displayed by number inside the circle, the depth of the circle’s color is proportional to the score, and the corresponding relationship between numbers and colors is shown by a color scale in lower panel.

The sequences of 2000 bp upstream of the start codon (ATG) were selected as *CqR2R3-MYB* promoters for *cis*-regulatory elements analysis. A total of 20 elements were identified in the promoter by PlantCARE software and the score of each element in each promoter is displayed digitally (Figure 4) [33]. The cis-acting elements are classified into four categories, including light response, plant hormone, plant growth, and stress response. The plant hormone group contains the most elements which are involved in the abscisic acid responsiveness (ABRE), gibberellin-responsive element (GARE-motif, P-box), salicylic acid responsiveness (TCA-element), auxin-responsive element (TGA-element), gibberellin-responsive (TATC-box), and MeJA-responsiveness (CGTCA-motif, TGACG-motif), suggesting that the *CqR2R3*-*MYB* genes are regulated by multiple hormones, similar to *R2R3-MYB* genes in many other plant species. These results indicate that *CqR2R3-MYB* genes are widely involved in various physiological and biochemical activities in plants, and responses to environmental stimuli and stress.

### 2.5. Expression Pattern of CqR2R3-MYB Family Genes in Quinoa Leaves under Saline–alkali Stress

**Figure 5:**
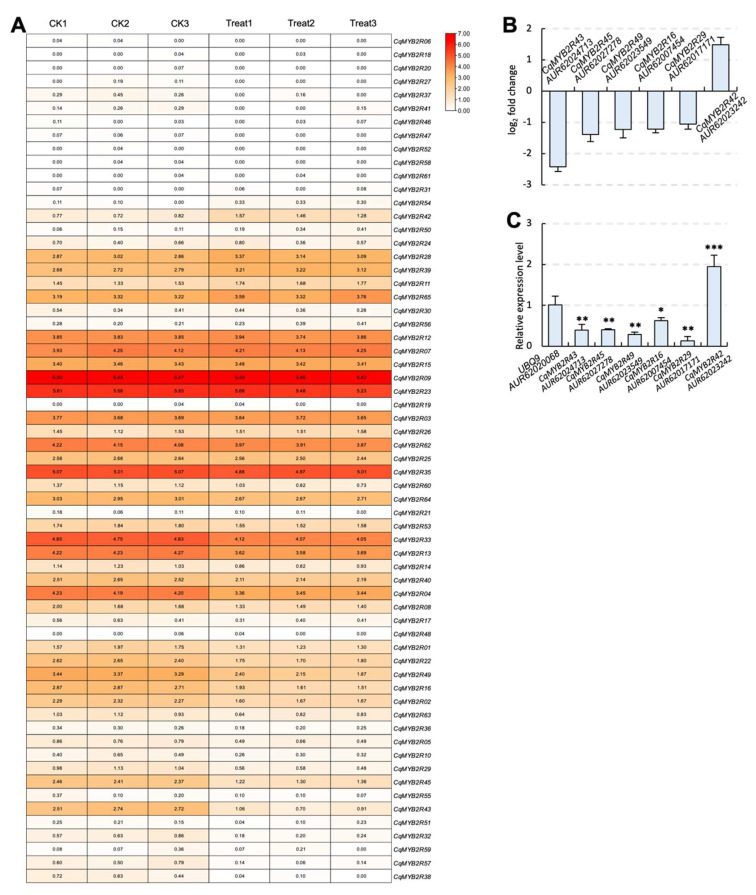
The expression pattern of *CqR2R3-MYB* genes. (**A**) The expression levels of all *CqR2R3-MYB* genes in quinoa leaves that had undergone saline–alkali stress. CK1, CK2, and CK3 are the three replicates of the CK group. Treat1, Treat2, and Treat3 are the three replicates of the carbonate-treatment group. (**B**) The log_2_ fold change of the six *CqR2R3-MYB* genes with the greatest changes in expression levels. (**C**) RT-qPCR validation of the *CqR2R3-MYB* genes in (**B**). *UBQ9* (AUR62020068) was used as an endogenous control. Three independent experiments per sample, three replicates per experiment. *, *p* < 0.05; **, *p* < 0.01; ***, *p* < 0.001; Student’s *t*-test.

To investigate the potential functions of the CqR2R3-MYB TFs under saline–alkali stress, we performed transcriptome analysis using quinoa leaves that had undergone 150 mM carbonate (100 mM NaHCO_3_ and Na_2_CO_3_ mixture) treatment. The results indicate that the six genes with the greatest changes in expression levels (log_2_ Fold Change (FC) > 1 or <−1) were *CqMYB2R43* (log_2_FC = −2.4181), *CqMYB2R45* (log_2_FC = −1.3827), *CqMYB2R49* (log_2_FC = −1.2214), *CqMYB2R16* (log_2_FC = −1.2183), *CqMYB2R29* (log_2_FC = −1.0544), and *CqMYB2R42* (log_2_FC = 1.48797) (Figure 5A,B). In order to verify the transcriptome data, we performed RT-qPCR analysis, and the results were consistent with the transcriptome data (Figure 5C). The significant changes of expression of these *CqR2R3-MYB* genes suggest that they were involved in saline–alkali stress response.

### 2.6. GO Enrichment Analysis of Differentially Expressed CqR2R3-MYB Genes

**Figure 6:**
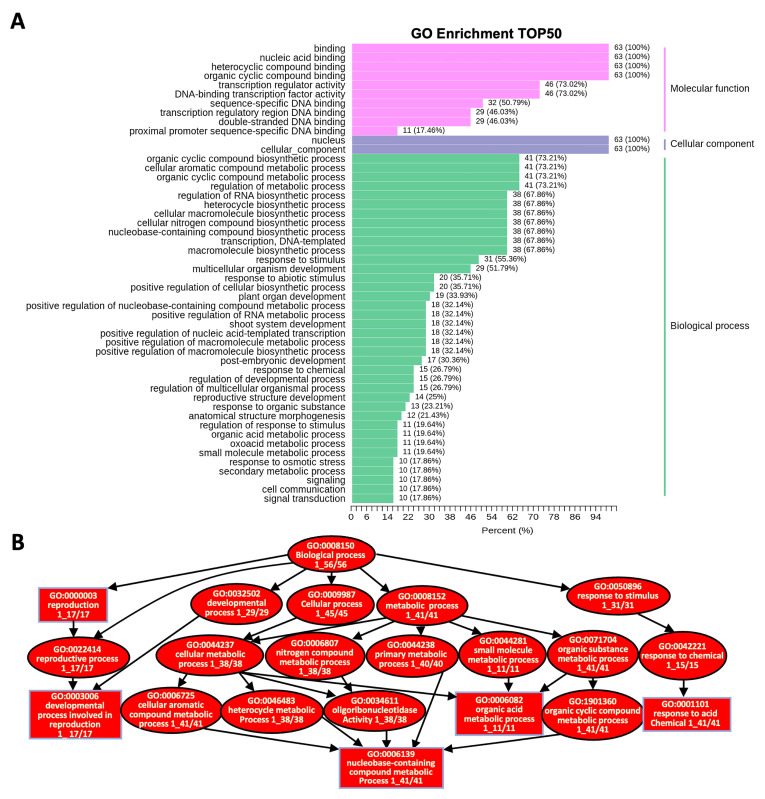
GO enrichment analysis of *CqR2R3-MYB* genes in quinoa leaves that had undergone saline–alkali stress. (**A**) Go enrichment analysis of TOP50 of *CqR2R2-MYB* genes. (**B**) The enriched GO terms in Biology Process of the differentially expressed *CqR2R2-MYBs*. Rectangles indicate the most significant terms. Rectangle and oval colors represent the relative significances, dark red indicates the most significant, *p* < 0.0001.

To achieve a broader functional characterization, the *CqR2R3-MYB* genes were subjected to GO enrichment analysis. As a result, the *CqR2R3-MYB* genes were categorized into 129 subcategories belonging to three main categories: 104 subcategories in Biological Processes (BP), 10 in Cellular Components (CC), and 15 in Molecular Functions (MF) (Figure 6A). The *CqR2R3-MYB* genes are widely involved in the biosynthesis and metabolic processes of metabolites (e.g., organic cyclic compound biosynthetic process (GO:1901362), cellular aromatic compound metabolic process (GO:0006725), and primary metabolic process (GO:0044238) etc.), development, and response to stimulus (Figure 6B). These results indicate that *CqR2R3-MYB* TFs are closely related to plant growth and development, and environmental stimuli response.

### 2.7. Subcellular Localization and Transcriptional Activation Activities of Four CqR2R3-MYBs

**Figure 7:**
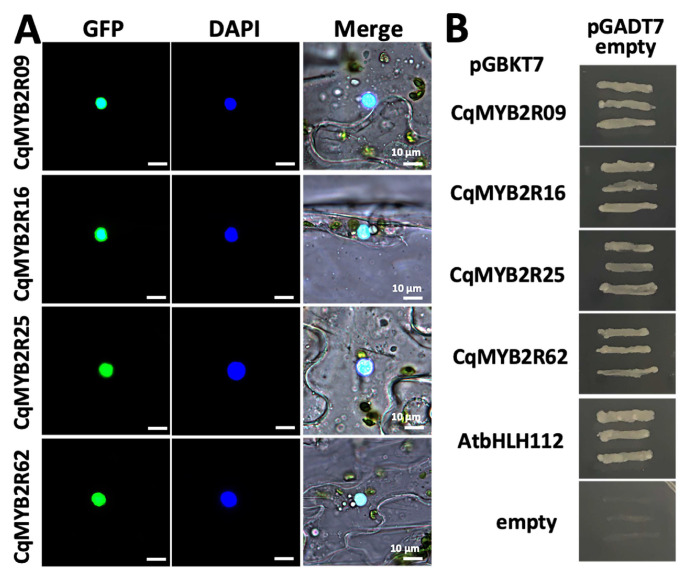
Determination of subcellular localization and autoactivation activity of CqR2R3-MYBs. (**A**) Subcellular localization of GFP-CqMYBs was detected by transient expression in tobacco leaves. The plasmids harboring *GFP-CqR2R3-MYBs* were transformed into GV3101 and infiltrated to tobacco leaves. The subcellular localization of GFP-CqR2R3-MYB fusion proteins were observed after DAPI staining. (**B**) Analysis of autoactivation activity. The *pGBKT7* plasmids harboring *GFP-CqR2R3-MYBs* were transformed with *pGADT7* vector into strain AH109, respectively, and grown on SD/-Leu/-Trp medium. The autoactivation activity was examined on SD/-Leu/-Trp/-His/-Ade medium. *pGBKT7* empty, a negative control; *AtbHLH112*, a positive control.

The subcellular location of CqR2R3-MYB TFs is related to their roles in the transcriptional regulatory network. Therefore, to validate their subcellular localization, we selected four CqR2R3-MYBs: CqMYB2R09, CqMYB2R16, CqMYB2R25, and CqMYB2R62, whose *Arabidopsis* homologous are related to salt stress response [13,34,35,36] (Supplementary Figure S2). Since the nucleus localization signals (NLSs) are in the middle of these TFs, we cloned the constructs with *GFP* fused to N-terminal of *CqR2R3-MYBs*. The transient assay analysis indicated that all the four GFP-CqR2R3-MYB fusion proteins exhibited nucleus localization which was identical to the prediction (Figure 7A). Then, we investigated the autoactivation activity of these four CqR2R3-MYBs. The recombinant plasmids, *pGBKT7-CqMYB2R09*, *pGBKT7-CqMYB2R16*, *pGBKT7-CqMYB2R25*, and *pGBKT7-CqMYB2R62*, were transformed into yeast strain AH109 with *pGADT7* vector, respectively. The transformants grew on SD/-Leu-Trp-His-Ade medium (Figure 7B), indicating that the four CqR2R3-MYBs have transcriptional activation activity. The above results indicate that CqR2R3-MYBs, CqMYB2R09, CqMYB2R16, CqMYB2R25, and CqMYB2R62 have the characteristics of TFs.

## 3. Discussion

### 3.1. Identification and Evolution of the Quinoa R2R3-MYB Gene Family

The *MYB* gene family is one of the largest families of transcription factor in plants, among which R2R3-MYB TF is the most abundant type [37]. With the development of sequencing technology and the improvement of genomic database of more species, the identification of *R2R3-MYB* genes is becoming more accurate; for example, 126 *R2R3-MYB* genes were identified in *Arabidopsis* [38], 244 in soybean [39], 157 in corn [40], and 88 in rice [41]. In this study, we systematically identified 65 *R2R3-MYB* members in the quinoa genome. Compared with *Arabidopsis thaliana*, the number of *R2R3-MYB* genes in quinoa is significantly lower than that in *Arabidopsis*. The number of *R2R3-MYB* genes in plants does not depend entirely on the size of the genome or the phylum of plants [32]. Variability in the number of *R2R3-MYB* genes might be attributed to the ploidy levels of species and gene duplication events during evolution [42].

The genome of quinoa is an allotetraploid (2n = 4x = 36) with an estimated genome size of approximately 1.5 Gb, but it contains only 65 *R2R3-MYB* genes, whereas the *Arabidopsis* genome is only 125 M, but contains 126 *R2R3-MYB* genes [29,43]. In fact, we have identified a total of 204 *MYB* genes in quinoa, of which 130 belong to the *R1-MYB* family, twice the number of *R2R3-MYB* genes. This is different from most species which have more *R2R3-MYBs* than *R1-MYBs* [44].

The quinoa R2R3-MYB TFs were phylogenetically clustered into 26 subgroups. The biological functions of most CqMYB2R TFs have not been characterized, whereas more than 90% of the CqMYB2R proteins are clustered with the known functions of *Arabidopsis* homologues [7]. For example, in S14, S15, and S21, the *Arabidopsis* members are involved in abiotic stress response and cell wall biosynthesis [45]. Consequently, phylogenetic analysis aids in the prediction of *CqMYB2R* gene functions.

Although some motifs are common to all members of CqMYB2Rs, there are significant differences in motif type, number, and alignment among different subfamilies, indicating a functional division. For example, motif 8 only appears in two proteins, motif 9 and motif 10 only appear in five proteins, respectively (Figure 3), suggesting a potential functional specificity of these TFs. Interestingly, although motif 1, motif 3, and motif 9 belong to the MYB domain, motif 9 only exists in five TFs (CqMYB2R04, CqMYB2R20, CqMYB2R24, CqMYB2R33 and CqMYB2R35) in the S28 subfamily, whereas motif 1 and motif 3 appear in all other TFs, emphasizing the functional specificity of the five TFs. All R2R3 motifs are consistently highly concentrated at the N-terminus, whereas the sequences at the C-terminus are variable, which endows CqR2R3-MYB TFs with a diversity of functions, e.g., activation or repression of transcription activities, etc. This is identical to the R2R3-MYB family proteins in many other species [11].

In short, this is the first identification and preliminary analysis of quinoa *R2R3-MYB* gene function, which provides basic information for further investigation.

### 3.2. Putative Functions of CqR2R3-MYB Transcription Factors

In this study, we analyzed TF characteristics of four CqMYB2R proteins, CqMYB2R09, CqMYB2R16, CqMYB2R25, and CqMYB2R62, whose *Arabidopsis* homologues are involved in saline–alkali stress response (Supplementary Figure S2). CqMYB2R09 is a homologous of AtMYB73, which is associated with the SOS pathway and thus involved in plant salt-stress response [30]. CqMYB2R16 is a homologous of AtMYB42s that can activate SOS2 expression [35]. CqMYB2R25 is a homologous of AtMYB49 which regulates the stratum corneum formation in *Arabidopsis* leaves in response to salt stress [13]. CqMYB2R62 is a homologous of AtMYB30 which regulates expression of *AOX1A* (alternative oxidase 1A) for plant resistance to salt stress [36]. All of the four CqMYB2R proteins presented strong autoactivation activities and nucleus localization, which is identical to the prediction. Therefore, the biological functions of these four CqMYB2R TFs deserve further investigation.

A total of 64 out of the 65 CqMYB2R proteins were predicted to have a nucleus localization, except for CqMYB2R35 with a dual localization of nucleus and cytoplasm. The R2R3-MYBs in other species also have this type TFs [37]. The function of CqMYB2R35 is also worth further research.

So far, the functional research of R2R3-MYB transcription factors mainly focused on the herbaceous model plant *Arabidopsis*, and the functions of about 80% of AtR2R3-MYBs have been demonstrated [44]. However, the research on quinoa is still in its early stages. There is currently no report on the functionality of CqR2R3-MYB family members. Identification of the CqMYB2Rs provides basic information for future functional research of CqR2R3-MYBs.

Our transcriptome analysis presented the expression profile of *CqMYB2Rs* in quinoa leaves that had undergone carbonate treatment (Figure 5). It revealed that six CqMYB2R TFs—CqMYB2R16, CqMYB2R29, CqMYB2R42, CqMYB2R43, CqMYB2R45 and CqMYB2R49—were involved in the saline–alkali stress response. Tomato MYB49, an R2R3-MYB TF, is a homologous of CqMYB2R49 (Supplementary Figure S2). *MYB49*-overexpressing tomato plants showed stronger tolerance to drought and salt stress [46]. Combined with its significant decrease in expression level under saline–alkali stress (Figure 5), CqMYB2R49 is likely to participate in stress response. It is worth investigating this point.

Usually, the expression of genes is determined by their regulatory elements in promoters. Many cis-acting elements in *CqMYB2R* promoters are widely involved in response to environmental stimuli and stress, such as MBS, LTR, and TC-rich repeat response to salt tress, ABRE and GCTGA-motif to phytohormone, and G-box and Box4 to light, etc. It is well known that R2R3-MYB TFs regulate flavonoid biosynthesis and other secondary metabolites synthesized in the Phenylpropane biosynthesis pathway [47]. The promoters of six *CqMYB2R* genes contain MBSI element, which acts as MYB binding sites involved in the regulation of flavonoid biosynthesis [48]. MBS is also a MYB binding site, and the binding is induced by drought stress [49]. TC-rich repeats are involved in defense and stress response [50]. In pear, ABRE-binding factor3 (PpABF3) promotes malate accumulation in response to salinity [51]. The JA signaling pathway participates in not only plant defense against abiotic and biotic stress, but also biosynthesis of flavonoids and anthocyanins, etc. [52,53]. Identification of JA-responsive elements (CGTCA-motif, TGACG-motif) suggests that some CqR2R3-MYB TFs are JA responsive. Interestingly, the Box4, G-box and ABRE in some genes have high scores (10–14), suggesting they may play more important roles in response to saline–alkali stress. However, the possibility of other genes participating in regulation cannot be ruled out.

R2R3-MYB transcription factors have been reported to play important roles in abiotic stress response in *Arabidopsis* and other species. For example, OsMYBR57 participates in the response to drought stress, and *OsMYBR57*-overexpressing transgenic rice had higher yields under drought stress [54]. *GmMYB14*-overexpressing transgenic soybean had better drought resistance and gained higher yields in high planting density under field conditions [55]. Our research also clarified that transcriptional levels of some *CqR2R3-MYB* genes altered significantly under saline–alkali stress, suggesting they may be involved in stress response. Further investigation will help to understanding their functions, which may provide target genes for molecular design breeding with the goal of generating stress resistant quinoa lines.

### 3.3. CqR2R3-MYB Genes in Cell Wall Biosynthesis

The R2R3-MYB gene family is closely related to Phenylpropane metabolism and secondary cell wall synthesis. R2R3-MYB TFs binds bHLH and WDR proteins to form MBW complexes regulating plant flavonoid biosynthesis [56]. The AtR2R3-MYBs in S5, S6, and S15 subfamilies, and the R/B-like bHLH TFs in IIIf subgroup, may synergistically regulate biosynthesis of flavonoid, anthocyanin, and proanthocyanidin (PA) [47]. The flavonoid biosynthesis-related cis-acting elements in CqR2R3-MYB promoters suggest the potential functions of CqMYB2Rs in S5, S6, and S15 subfamilies.

In *Arabidopsis*, a significant number of *R2R3-MYB* genes are involved in cell wall biosynthesis, e.g., MYB20, MYB42, MYB43, and MYB85 can regulate phenylalanine and lignin biosynthesis during secondary cell wall formation [57]. They can bind to the promoters of *ADT6* (Arogenate dehydratase 6) and *HTC* (quinate hydroxycinnamoyl transferase) and activate their expression [58]. These MYB TFs also directly activate expression of the genes for lignin and phenylpropanoid biosynthesis during secondary wall formation [57]. Whether the quinoa homologous genes of the *Arabidopsis R2R3-MYB* genes also have these functions is a scientific question worth exploring.

In summary, we identified 65 CqR2R3-MYB family genes, analyzed their protein and gene structure and expression pattern under saline–alkali stress, and verified their subcellular localization and transcriptional activation abilities. Our study provides preliminary evidence that some CqR2R3MYB transcription factors may play important roles in stress response in quinoa.

## 4. Materials and Methods

### 4.1. Plant Materials, Growth Conditions, and Stress Treatments

Quinoa (Jiaqi #3) (provided by Jiaqi Agricultural Technology Co., Ltd., Taiyuan, Shanxi, China) plants grew under a 16 h light/8 h dark cycle at 22 °C. Two-week-old quinoa seedlings were treated with a solution containing 100 mM Na_2_CO_3_:NaHCO_3_ = 1:9 once every 5 days, three times in total. The control group was treated with water. The leaves were harvested randomly five days after the third treatment, then sent for RNA sequencing. The datasets used in the current study are deposited in NCBI Sequence Read Archive (SRA) Database as accession numbers PRJNA972512.

### 4.2. Genome-Wide Identification of R2R3-MYB Family Members in Quinoa (Chenopodium quinoa Willd.)

From Ensemble Plants (http://plants.ensembl.org/index.html, accessed on 23 March 2023), we downloaded the genome file and GFF3 file of quinoa (http://plants.ensembl.org/index.html, accessed on 23 March 2023), and from TAIR (https://www.Arabidopsis.org/, accessed on 23 March 2023), we downloaded *Arabidopsis* R2R3-MYB protein sequences.

### 4.3. Bioinformatics Analysis of Quinoa R2R3-MYB Family Genes

#### 4.3.1. Phylogenetic Analysis

The phylogenetic tree of quinoa and *Arabidopsis* R2R3-MYB families was constructed based on multiple protein sequence alignment using MAGA 11 software [59] and the maximum-likelihood model (bootstrap is 1000). iTOL (http://itol.embl.de/, accessed on 25 March 2023) website was used to optimize the evolutionary tree.

#### 4.3.2. The Physicochemical Properties, Conserved Motif Analysis

The protein length, isoelectric point (pI), and molecular weight (MW) were predicted using ExPASy website (https://www.expasy.org/, accessed on 25 March 2023) [60].

The conserved motifs were retrieved by searching MEME website (https://meme-suite.org/meme/doc/meme.html, accessed on 26 March 2023) [61]. The maximum retrieval value was set to 10, and the other parameters were default. InterProScan software (InterProScan 5.62-94.0, EMBL, Heidelberg, Germany) was used to annotate the motifs.

#### 4.3.3. Gene Structure and Cis-Acting Element Analysis

The gene structure of the *R2R3-MYB* family genes was analyzed using TBtools combined with the GFF3 gene annotation data, and to plot the exon–intron diagram.

The 2000 bp sequences upstream of start codon of *R2R3-MYB* genes were screened using TBtools and used as promoter sequences for analysis. The plantCARE (https://bioinformatics.psb.ugent.be/webtools/plantcare/html/, accessed on 2 April 2023) [33] was used to investigate the cis-acting elements in promoters to predict the regulatory roles of genes in stress responses.

### 4.4. RT-qPCR Validation

The total RNA used for RT-qPCR (reverse transcription quantitative PCR) was the same sample as that for RNA sequencing. RT-qPCR was performed following the manufacturer’s instructions for Ultra SYBR Mixture (Low ROX) on the ABI7300 real-time PCR system (Applied Biosystems, Waltham, MA, USA). *UBQ9* (AUR62020068) was used as the reference gene for normalizing mRNA transcription [62]. The relative expression level was calculated by the 2^−∆∆CT^ method [63]. All RT-qPCR analyses were set with 3 technical repeats. The primers are listed in Supplemental Table S1.

### 4.5. Subcellular Localization of CqR2R3-MYBs

The coding region of *CqMYB2R09*, *CqMYB2R16*, *CqMYB2R25,* and *CqMYB2R62* were amplified and ligated into *pCAMBIA1300* vector with an enhanced green fluorescent protein (GFP) fused in N-terminal. The plasmids were transformed into *Agrobacterium tumefaciens* (GV3101) and infiltrated to tobacco leaves. After growing under light conditions for 48 h, the tobacco leaves were stained with DAPI [0.1 M sodium phosphate (pH 7.0), 1 mM EDTA, 0.1% Triton X-100 (*v*/*v*), and 0.5 mg/mL DAPI], then the subcellular localization of GFP-CqR2R3-MYB fusion proteins were observed by Leica fluorescence microscope (DM4 B, Wetzlar, Germany) (GFP, excitation 488 nm, emission 507 nm; DAPI, excitation 340 nm, emission 488 nm).

### 4.6. Transcriptional Activation Assay

For transcriptional activation assay, the coding region of *CqMYB2R09*, *CqMYB2R16*, *CqMYB2R25,* and *CqMYB2R62* were amplified and fused in-frame downstream of the GAL4 DNA binding domain in *pGBKT7* vector. The constructs were transformed with pGADT7 vector into strain AH109 (*S. cerevisiae*) (Clontech), respectively, and grown on SD/-Leu/-Trp medium. The autoactivation activity was examined on SD/-Leu/-Trp/-His/-Ade medium. The empty *pGBKT7* vector and *pGBKT7-AtbHLH112* construct were used as negative and positive controls, respectively.

## 5. Conclusions

In this study, we identified 65 R2R3-MYB genes in the quinoa genome and analyzed their physicochemical properties, evolutionary relationships, conserved domains, and motifs in proteins, gene structure, and cis-regulatory elements in promoters. We also confirmed the subcellular localization and transcriptional activation activity of four CqMYB2R TFs whose *Arabidopsis* homologues are involved in salt stress response. The results indicate that the TF characteristics are conserved in CqR2R3-MYB family proteins. The transcriptome analysis reveals that some CqR2R3-MYBs are important for response to saline–alkali stress. Our study provides basic information of R2R3-MYB family TFs in quinoa for future functional research.

## Figures and Tables

**Table 1:** Basic information of CqR2R3-MYB proteins. GRAVY, grand average of hydropathicity.

Gene ID	Name	Length (aa)	Molecular Weight (KDa)	pI	Instability Index	Ali-Phatic Index	GRAVY	Localization Predictor
AUR62000115	CqMYB2R01	306	33.72	5.4	50.02	61.5	−0.606	nucleus
AUR62000244	CqMYB2R02	556	60.79	5.18	57.53	65.43	−0.619	nucleus
AUR62000484	CqMYB2R03	979	110.49	5.11	49.31	70.91	−0.921	nucleus
AUR62001688	CqMYB2R04	308	34.54	6.72	45.02	62.08	−0.776	nucleus
AUR62001998	CqMYB2R05	341	38.48	6.3	44.53	78.97	−0.633	nucleus
AUR62002080	CqMYB2R06	360	39.79	6.86	46.36	77.83	−0.506	nucleus
AUR62002136	CqMYB2R07	235	26.62	4.23	60.72	65.53	−0.586	nucleus
AUR62003804	CqMYB2R08	338	38.12	6.23	43.85	77.93	−0.626	nucleus
AUR62003939	CqMYB2R09	302	32.84	8.27	55.99	65.23	−0.526	nucleus
AUR62004043	CqMYB2R10	322	36.11	6	53.7	67.61	−0.635	nucleus
AUR62004326	CqMYB2R11	299	33.91	6.27	58.66	67.22	−0.709	nucleus
AUR62004628	CqMYB2R12	324	35.11	8.7	39.71	67.47	−0.594	nucleus
AUR62005744	CqMYB2R13	461	51.94	7.14	54.91	73.64	−0.691	nucleus
AUR62006590	CqMYB2R14	551	60.21	5.15	59.05	65.5	−0.602	nucleus
AUR62006826	CqMYB2R15	979	110.30	5.18	49.52	71.71	−0.889	nucleus
AUR62007454	CqMYB2R16	304	34.04	4.94	50.21	73.78	−0.701	nucleus
AUR62007558	CqMYB2R17	322	36.26	6.01	55.41	64.57	−0.692	nucleus
AUR62008199	CqMYB2R18	456	51.25	5.78	42.26	69.47	−0.693	nucleus
AUR62008324	CqMYB2R19	361	40.00	5.94	50.63	61.39	−0.711	nucleus
AUR62008966	CqMYB2R20	255	29.02	5.23	65.49	61.22	−0.687	nucleus
AUR62011751	CqMYB2R21	287	33.03	5.7	54.07	67.28	−0.771	nucleus
AUR62011870	CqMYB2R22	286	32.26	5.61	53.48	63.11	−0.772	nucleus
AUR62013046	CqMYB2R23	88	10.10	9.1	53.32	77.73	−0.716	nucleus
AUR62014537	CqMYB2R24	199	22.83	4.58	63.06	61.26	−0.632	nucleus
AUR62014700	CqMYB2R25	361	40.99	5.56	46.85	61.61	−0.739	nucleus
AUR62014701	CqMYB2R26	389	43.66	5.85	45.88	74.24	−0.713	nucleus
AUR62014702	CqMYB2R27	289	32.55	6.96	50.96	80.66	−0.55	nucleus
AUR62015573	CqMYB2R28	240	27.08	4.24	59.92	70.29	−0.588	nucleus
AUR62017171	CqMYB2R29	316	35.16	6.76	37.49	69.49	−0.618	nucleus
AUR62018324	CqMYB2R30	275	31.21	6.45	47.97	71.64	−0.737	nucleus
AUR62018693	CqMYB2R31	244	27.54	6.23	36.97	79.18	−0.609	nucleus
AUR62019989	CqMYB2R32	154	17.67	10.18	55.73	70.26	−0.929	nucleus
AUR62020094	CqMYB2R33	308	34.54	6.72	46.45	63.34	−0.783	nucleus
AUR62020426	CqMYB2R34	123	13.26	9.51	30.03	85.61	−0.354	nucleus
AUR62020972	CqMYB2R35	321	35.24	8.89	44.35	72.02	−0.539	nucleus/cytoplasm
AUR62021197	CqMYB2R36	221	25.45	6.31	51.58	63.53	−0.76	nucleus
AUR62021199	CqMYB2R37	283	31.59	8.3	56.05	63.78	−0.639	nucleus
AUR62022338	CqMYB2R38	322	36.48	9.58	56.34	52.14	−0.926	nucleus
AUR62022709	CqMYB2R39	322	34.98	8.77	38.89	69.44	−0.534	nucleus
AUR62022815	CqMYB2R40	424	46.38	5.33	58.83	68.8	−0.537	nucleus
AUR62022912	CqMYB2R41	257	28.42	7.06	39.9	70.82	−0.866	nucleus
AUR62023242	CqMYB2R42	210	23.98	8.86	51.63	72.1	−0.818	nucleus
AUR62023549	CqMYB2R43	329	36.94	6.46	45.5	70.82	−0.696	nucleus
AUR62024595	CqMYB2R44	311	35.52	6.86	53.94	76.17	−0.496	nucleus
AUR62024713	CqMYB2R45	295	33.23	6.22	51.23	63.83	−0.745	nucleus
AUR62025096	CqMYB2R46	601	68.36	8.94	37.19	77.24	−0.598	nucleus
AUR62025146	CqMYB2R47	241	27.83	5.55	42.42	78.13	−0.532	nucleus
AUR62025185	CqMYB2R48	290	33.13	9.15	60.24	77.97	−0.746	nucleus
AUR62027278	CqMYB2R49	306	34.27	4.8	49.3	73.63	−0.705	nucleus
AUR62028989	CqMYB2R50	289	32.96	9.05	61.29	81.97	−0.697	nucleus
AUR62030563	CqMYB2R51	328	36.76	6.15	48.05	74.6	−0.608	nucleus
AUR62030594	CqMYB2R52	322	36.27	6.05	42.53	65.47	−0.641	nucleus
AUR62032801	CqMYB2R53	300	33.56	8.75	76.97	55.67	−0.755	nucleus
AUR62033319	CqMYB2R54	299	33.76	6.1	60.77	67.26	−0.671	nucleus
AUR62033340	CqMYB2R55	299	33.76	6.1	60.77	67.26	−0.671	nucleus
AUR62033728	CqMYB2R56	287	33.21	6.18	47.29	71.67	−0.752	nucleus
AUR62034368	CqMYB2R57	282	32.15	9.49	56.86	55.39	−0.983	nucleus
AUR62034976	CqMYB2R58	362	40.68	6.72	55.92	72.98	−0.707	nucleus
AUR62036035	CqMYB2R59	206	22.93	9.17	31.01	69.71	−0.568	nucleus
AUR62037317	CqMYB2R60	351	40.10	5.16	60.18	70.57	−0.778	nucleus
AUR62039339	CqMYB2R61	373	41.99	6.41	49.48	76.81	−0.659	nucleus
AUR62039812	CqMYB2R62	355	40.16	6.73	62.19	56.34	−0.946	nucleus
AUR62040017	CqMYB2R63	333	37.29	6.75	47.8	65.86	−0.803	nucleus
AUR62041016	CqMYB2R64	468	52.18	8.29	52.9	65.66	−0.709	nucleus
AUR62041339	CqMYB2R65	369	42.09	9	64.54	71.17	−0.801	nucleus

## Data Availability

All data are presented in this article.

## Supplementary Materials

The following supporting information can be downloaded at: https://www.mdpi.com/article/10.3390/ijms24119132/s1.
