# Supplementary material for: Genome-Wide Identification and Analysis of R2R3-MYB Genes Response to Saline–Alkali Stress in Quinoa
Source: Int J Mol Sci. 2023 May 23;24(11):9132. doi: 10.3390/ijms24119132 (PMC10252952; doi:10.3390/ijms24119132)
Supplement: Supplementary file 1 [file ijms-24-09132-s001.zip › supplement table1.pdf]

Supplementary Table 1. All primer sequences used in this study

Gene Name	Sequence (5'-3')	experiment
43-CqMYB2R16-F	GACGAGCTGTACAAGGGCGCGCCATCTAGAATGGGACGACAACCTTG	Subcellular Localization
43-CqMYB2R16-R	CCGCTCTAGAACTAGTTAATTAAGGTACCTTGCTTGTTATCCATTTCTA ACACATCC	Subcellular Localization
43-CqMYB2R25-F	GACGAGCTGTACAAGGGCGCGCCATCTAGAATGGGTAGAGCACCTTG	Subcellular Localization
43-CqMYB2R25-R	CCGCTCTAGAACTAGTTAATTAAGGTACCTCAGTTGGTTTGAGCTAAC A	Subcellular Localization
43-CqMYB2R62-F	GACGAGCTGTACAAGGGCGCGCCATCTAGAATGGGAAGGAGTCCGTG TTG	Subcellular Localization
43-CqMYB2R62-R	CCGCTCTAGAACTAGTTAATTAAGGTACCTCATTCAAAATCAGCAGTT TCTCGCAAGG	Subcellular Localization
43-CqMYB2R09-F	GACGAGCTGTACAAGGGCGCGCCATCTAGAATGAACAGGAAAGAAGT GGACC	Subcellular Localization
43-CqMYB2R09-R	CCGCTCTAGAACTAGTTAATTAAGGTACCCTACTCAATTTTTCCGATTC CAATTCTACTC	Subcellular Localization
BD-CqMYB2R16-F	ATGGCCATGGAGGCCGAATTCTCTAGAATGGGACGACAACCTTG	Transcriptional Activation Activity
BD-CqMYB2R16-R	CCGCTGCAGGTCGACGGATCCGGTACCTTGCTTGTTATCCATTTCTAAC ACATCC	Transcriptional Activation Activity
BD-CqMYB2R25-F	ATGGCCATGGAGGCCGAATTCTCTAGAATGGGTAGAGCACCTTG	Transcriptional Activation Activity
BD-CqMYB2R25-R	CCGCTGCAGGTCGACGGATCCGGTACCTCAGTTGGTTTGAGCTAACA	Transcriptional Activation Activity
BD-CqMYB2R62-F	ATGGCCATGGAGGCCGAATTCTCTAGAATGGGAAGGAGTCCGTGTTG	Transcriptional Activation Activity
BD-CqMYB2R62-R	CCGCTGCAGGTCGACGGATCCGGTACCTCATTCAAAATCAGCAGTTTC TCGCAAGG	Transcriptional Activation Activity
BD-CqMYB2R09-F	ATGGCCATGGAGGCCGAATTCTCTAGAATGAACAGGAAAGAAGTGGA CC	Transcriptional Activation Activity
BD-CqMYB2R09-R	CCGCTGCAGGTCGACGGATCCGGTACCCTACTCAATTTTTCCGATTCCA ATTCTACTC	Transcriptional Activation Activity
qPCR-UBQ9-F	CAATGTCTACTACAACGAAGCG	qPCR
qPCR-UBQ9-R	CAGACTGACCAAACACAAAGTT	qPCR
qPCR-CqMYB2R16 -F	ACGGTCATTAATTCAATGTCGGAGG	qPCR
qPCR-CqMYB2R16 -R	CGTCGTCATCCCATAAGTAACTCAT	qPCR
qPCR-CqMYB2R29 -F	TTGCGAGAAGGAAAACGTAAAG	qPCR
qPCR-CqMYB2R29 -R	CCATGCTTAAGGTCAGGTCTTA	qPCR
qPCR-CqMYB2R42 -F	TCCTATCGCGATTTCATCTGAA	qPCR
qPCR-CqMYB2R42 -R	CAAAGGCGAGTTTAAGGTTCAA	qPCR
qPCR-CqMYB2R43 -F	AAGAAACGCCTCAAAGAGAAAC	qPCR
qPCR-CqMYB2R43 -R	ATGAGCTTGAATACTCGATCGT	qPCR
qPCR-CqMYB2R45 -F	TTGTAGCAAGGAAAATGTGGTG	qPCR
qPCR-CqMYB2R45 -R	CAACACTTCCATCCAGAAATCG	qPCR
qPCR-CqMYB2R49-F	ATTCTGATATAAGCACGGCTGA	qPCR
qPCR-CqMYB2R49-R	TGCTATTAGCTGGATCGCTATT	qPCR
