# Supplementary material for: Genome-Wide Identification and Analysis of R2R3-MYB Genes Response to Saline–Alkali Stress in Quinoa
Source: Int J Mol Sci. 2023 May 23;24(11):9132. doi: 10.3390/ijms24119132 (PMC10252952; doi:10.3390/ijms24119132)
Supplement: Supplementary file 1 [file ijms-24-09132-s001.zip › Supplementary Figures.pdf]

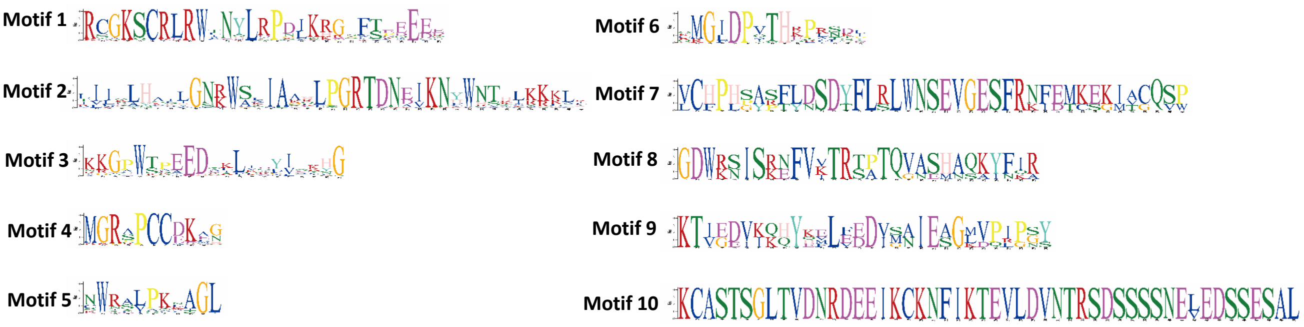

Supplementary Figure 1. Sequence analysis of conservative motifs in CqR2R3-MYB proteins. Different color represents different amino acid residues, and larger font sizes represent higher frequencies.

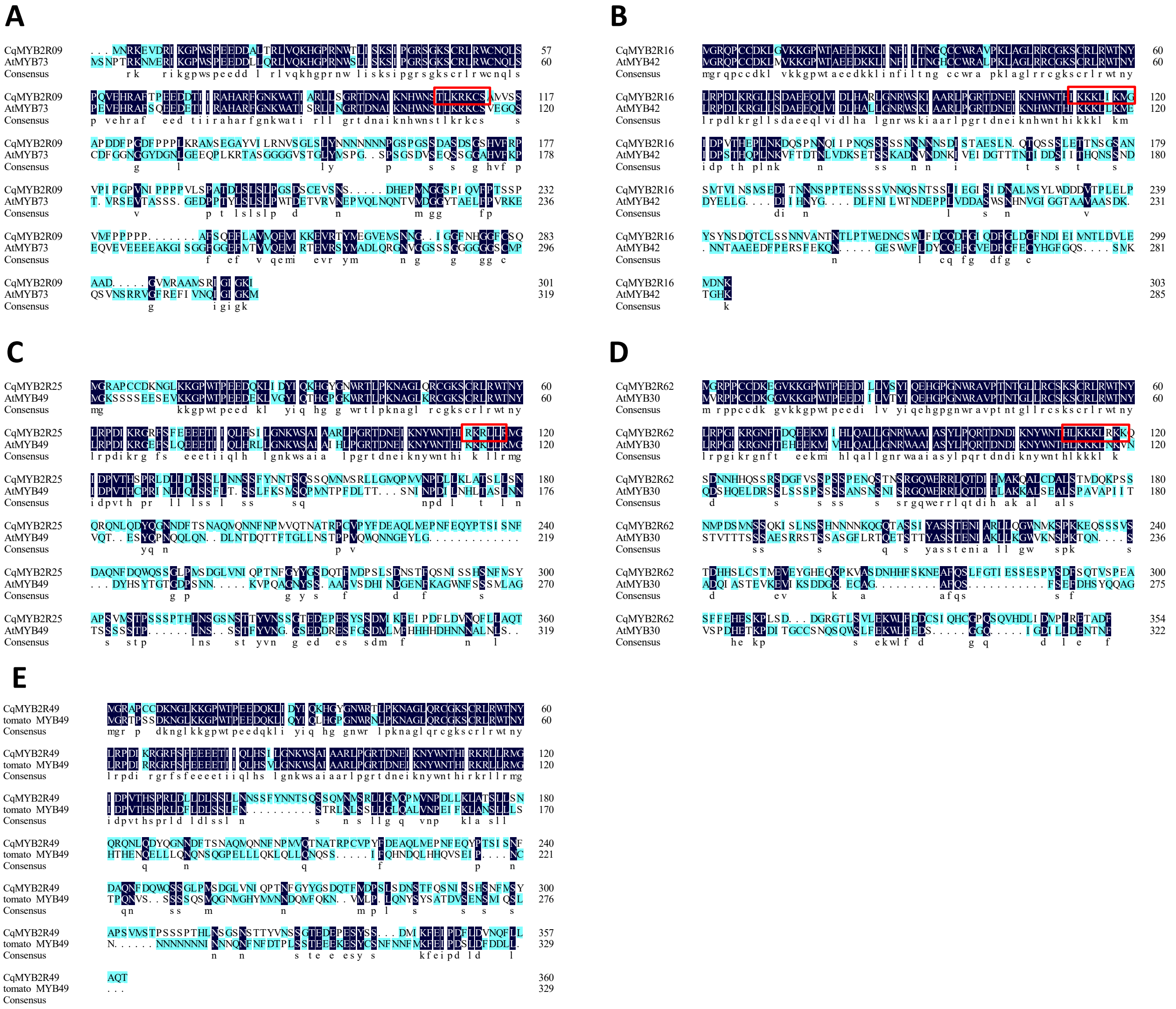

Supplementary Figure 2. Multi-sequence alignment of R2R3-MYB proteins of *Arabidopsis* and quinoa**.** Amino acid sequence alignment of CqMYB2R09 and AtMYB73 **(A)**, CqMYB2R16 and AtMYB42 **(B)**, CqMYB2R25 and AtMYB49 **(C)**, CqMYB2R62 and AtMYB30 **(D),** and CqMYB2R49 and tomato MYB49 **(E)**. Black background represents sequence identities = 100%. Red box highlights nuclear localization signals (NLSs).
